# A multivariate analysis to identify the relationship between sociodemographic differences and examination performance in UK postgraduate medical examinations

**DOI:** 10.1177/01410768251380980

**Published:** 2025-11-03

**Authors:** Ricky Ellis, Andy Knapton, Jane Cannon, Amanda J Lee, Jennifer Cleland

**Affiliations:** 1 Institute of Applied Health Sciences, University of Aberdeen, Aberdeen, AB25 2ZD, UK; 2 General Medical Council, General Medical Council, Education & Standards, Regent’s Place, London NW1 3AW, UK; 3 Institute of Applied Health Sciences, University of Aberdeen, Aberdeen, AB25 2ZD, UK; 4 Lee Kong Chian School of Medicine, Nanyang Technological University Singapore, i1 Mandalay Road, Singapore 308232

**Keywords:** Medical careers, medical education, quantitative research, statistics, research methods

## Abstract

**Objectives:**

Significant differences in group-level performance have been identified in UK postgraduate medical examinations. However, few examinations have been investigated independently, and those that have, focus on a limited number of sociodemographic factors. This study addresses these gaps by identifying predictors of success in each UK postgraduate medical examination, accounting for prior academic attainment and other sociodemographic differences.

**Design:**

Retrospective cohort study.

**Setting:**

Secondary care.

**Participants:**

Anonymised pass/fail data at the first attempt held within the General Medical Council Database were analysed for all candidates (UK medical school graduates [UKG] and international, non-UK graduates [IMG]) attempting a postgraduate examination between 2014 and 2020.

**Main outcome measures:**

Multivariate logistic regression models identified independent predictors of success at each postgraduate examination.

**Results:**

During the study period, 180,890 examination first-attempts were made by candidates, and 121,745 (67.3%) passed at their first attempt. Multivariate regression models revealed that place of primary qualification, gender, age, ethnicity, religion, sexual orientation, disability and LTFT status were all statistically significant independent predictors of success or failure in written and clinical examinations. The strongest independent predictors of failing written and clinical examinations were being an IMG, being from a minority ethnic background and having a registered disability.

**Conclusions:**

This was the largest study to date investigating independent predictors of outcomes at each UK postgraduate medical examination. Significant differences in pass rates were seen according to sociodemographic differences in each examination. These data can be used by Medical Royal Colleges, the GMC and training institutions to guide more granular research and future interventions.

## Introduction

Significant differences in group-level performance have been identified in UK postgraduate medical examinations. Several groups are known to have significantly lower pass rates than others at some high-stakes postgraduate examinations. These different levels of success and failure are not due to learner deficit or sociodemographic characteristics; rather, assessments are windows through which we see the impact of cumulative unequal social and educational opportunities, historical bias, minoritisation and discrimination.^1–6^ Importantly, these patterns are not unique to the UK: they are also seen in postgraduate medical examinations in other countries, such as the USA,^7^ and thus the method and outcomes of this paper will be of interest to those involved in medical and education and training in many different contexts.

Failure at high-stakes examinations used as gatekeeping assessments has a considerable impact on the lives and career progression of doctors. In recent years, increasing attention has been given to identifying and addressing this differential attainment (DA), prioritising equity and fairness within medical training and assessment to create a diverse and inclusive medical workforce known to positively impact patient health outcomes.^8,9^ In the UK, public authorities such as universities, Medical Royal Colleges, the National Health Service (NHS) and the General Medical Council (GMC) have a legal duty to address differences between groups with and without specific characteristics protected by the Equality Act 2010. There is a mandate for organisations within the UK^10–13^ and in other countries^14,15^ to address DA and ensure that any policy and practice changes have a positive impact on minoritised groups.

Our position is that differences in performance at the postgraduate examination level are likely to be related to continued structural and systemic inequalities as potential barriers to success. However, to successfully address DA and close the attainment gap, there needs to be a much more granular understanding of attainment patterns in assessments used throughout the medical training journey. This understanding will help focus change efforts and the provision of support/resources to trainees from underperforming groups. However, until now, most of the work on DA has been limited in scope to one specialty examination,^16–23^ or one type of assessment (written or clinical),^24–27^ and very few direct comparisons across specialties exist.^28,29^ Similarly, much of the work has used univariate and descriptive analyses to look at specific protected characteristics. While useful, these analyses do not take into account the relationship between variables, their independence from each other, and cannot delineate whether group-level differences in performance are largely related to characteristics or ability (with the latter indicated by prior academic attainment). This restricted scope limits the understanding of the intersectionality of different characteristics in relation to progression.^30^

To address these gaps in the literature and provide a more granular understanding of attainment patterns, this study aimed to use multivariate (MV) analyses to identify sociodemographic differences that predict success or failure in each of the postgraduate medical examinations used in the UK.

In the UK, and indeed in many other countries, group-level differences in education performance and progression are seen early in life,^31^ and such differential performance persists into adulthood^32^ with later performance reflecting early performance, including in medical education.^33,34^ Thus, we adjusted for prior attainment to attempt to isolate the relationship between sociodemographic factors and postgraduate examination success.

## Methods

After consultation with key stakeholders, we adopted the same terminologies as those used by the GMC, within the data held by the GMC (henceforth the GMC database) and by the wider literature at the time of writing to enable contextual comparison. The use of the term ‘minority group’ refers to all groups minoritised within the UK medical environment, whether by underrepresentation, disadvantage, or DA in training and assessment outcomes. This aligns with the definition by Selvarajah et al.^35^: ‘individuals and populations, including numerical majorities, whose collective cultural, economic, political and social power has been eroded through the targeting of identity in active processes that sustain structures of hegemony’. We recognise the power of words and appreciate that some terms used throughout this paper may not be preferred by all, may not reflect the identities or lived experience of individuals and are likely to change over time, but it is necessary to have a starting point.

### Study design

This retrospective cohort study used anonymised data held in the GMC database. Anonymised data were extracted by the GMC data project manager for all candidates (UK and overseas graduates, candidates with and without a national training number (NTN) at the time of sitting an examination) who attempted any UK post-graduate medical examination between 2014 and 2020 (prior to the changes made to examinations during the COVID-19 pandemic).

### Data aggregation

Rules for handling and aggregating data were established before data extraction, and access was granted to the research team. In line with the Higher Education Statistics Agency data standards (www.hesa.ac.uk), all counts presented have been rounded to the nearest 5 to ensure person-level anonymity. Examination pass/fail at the first attempt, as recorded in the GMC database, was used as the outcome measure, as this is known to be a strong predictor of later success in medical examinations.^36^

Post-graduate medical examinations vary significantly in terms of timing, format, number of components and delivery. In terms of timing, a large proportion of postgraduate medical examinations can be broken down into Membership examinations (e.g. Membership of the Royal College of Physicians or Surgeons), which are usually taken in the early years of postgraduate training (see later for exceptions) and are typically a prerequisite to progress into higher levels of training, and Fellowship examinations (e.g. Fellowship of the Royal College of Physicians or Surgeons), which are taken at the end of training/residency, and are used as an indicator of readiness to complete training and seek Consultant status. Other types of examination include, but are not limited to, Specialty Certificate Examinations (SCE) (e.g. Acute Medicine, Geriatric Medicine), which test specialty knowledge and are generally taken later on in a training program. A detailed description of each examination and its place within medical training pathways lies outside the scope of this paper, but can be found online.^37^

In terms of format and components, where relevant, examinations were aggregated into their written components and Clinical/Objective Structured Clinical Examination (OSCE)/Viva voce (herein described as ‘clinical’ examination) components. Given the aggregation of some examination components, the names of some examinations were altered. Details are provided in the examination key in Supplemental Table 1. We assumed that each candidate passed all component modules of each Part (written or clinical) of the examination at the same time, as is the case for the vast majority of candidates, as suggested by published exam data, and the date of the first attempt at the last component of each part (written or clinical) was used as the examination date. Examinations with fewer than 200 recorded cases were excluded to ensure sufficient statistical power to provide meaningful analyses. This meant the following medical examinations were excluded from the MV statistical analyses: Diploma in Pharmaceutical Medicine, Membership of the Faculty of Occupational Medicine, Membership of the Faculty of Sexual and Reproductive Health, Diploma in Otolaryngology – Head and Neck Surgery written, Faculty of Public Health and several specialty certificate examinations, including: Neurology, Infectious Diseases, Medical Oncology and the European Board of Gastroenterology and Hepatology Examination.

Self-declared ethnicity and religion were aggregated to align with previous GMC publications and data analyses on DA. This enabled comparison of the current analyses with previous ones. Age was dichotomised into either ⩽29 years old or >29 years old at the time of taking the examination; a cut-off designed to capture those who did a medical degree as an undergraduate and had limited time out of training (e.g. for maternity leave or a year out between the early, generic years of training [the Foundation Programme] and entering specialty training) versus more mature candidates who may have taken more time out of training, undertaken medicine as a graduate or had several years in a different career before starting medicine. Those missing less than full-time training (LTFT) data were assumed to work full-time, as the percentage of LTFT trainees in the study cohort corresponded with that in recent workforce reports.^38^ Data for other demographic variables are presented as held in the GMC database.

### Adjustment for prior academic attainment

Almost all UK medical school graduates (henceforth referred to as UKGs) in the GMC database have linked Universities and Colleges Admissions Service (UCAS) Tariff scores. The UCAS Tariff is used as an indicator of prior academic attainment at the point of selection into medical school. It is a means of allocating points to post-16 qualifications (e.g. A-Levels, Highers and other high school exit examinations), based on a simple mathematical model that uses a qualification size and grading scale to generate a total number of points. High school performance has been shown to correlate with success in postgraduate medical examinations.^33,34,39^ Information regarding how UCAS Tariff scores are calculated can be found at https://www.ucas.com/.

International medical graduates (IMGs), those who have obtained their primary medical qualification (PMQ) in a country other than the UK (including candidates graduating from schools in the European Economic Area), do not generally have a UCAS tariff. Thus, the outcome of the Professional and Linguistic Assessments Board (PLAB) examination was used for IMGs instead. The PLAB aims to ‘check that IMGs know and can do the same as a doctor starting the second year of their Foundation Programme training in the UK’. Successful completion of the PLAB is required to register for a GMC license to practice medicine and performance correlates with later success at postgraduate examinations.^29,40,41^ Individual-linked UCAS Tariff scores (for UKGs) and PLAB scores (for IMGs) relative to the pass mark were therefore converted to z-scores (to take account of changes to pass marks between each examination diet within the study period) and used as measures of prior academic attainment.

In summary, the use of prior academic attainment indicators, UCAS Tariff scores (for UKGs) or PLAB scores (for IMGs) provided a numerical measure of prior academic attainment for each candidate within the dataset.

### Statistical analysis

Univariate analysis was used to determine any associations with first-attempt examination outcomes. Missing data (including where data were not declared by individuals during data collection exercises) were excluded from regression analyses, with all analyses performed on a complete-case basis, and the total cohort used in each analysis (*N*) is stated in each table. There were very little first-language data available for IMGs (<0.1%), so this variable was excluded from regression analyses. Logistic regression (LR) models were created for the written and clinical components of each postgraduate examination separately using backwards conditional MV regression analyses. Only variables that remained significant in the final MV model after adjusting for all other variables are presented. Separate MV models were created for each examination. We acknowledge that running multiple statistical tests potentially reduces the statistical power. However, the application of a correction for multiple testing (such as the Bonferroni correction) is known to be conservative, also potentially leading to loss of statistical power and an increased risk of type II errors. Presenting confidence intervals allows the reader to assess the precision of the effect sizes. All analyses were conducted using SPSS^®^ for Windows v24.0 (IBM Corp, Armonk, NY, USA).

## Results

Between 2014 and 2020, 180,890 first attempts were made at UK postgraduate medical examinations by candidates who were either UKG or IMG (including candidates with and those without national training numbers). A total of 121,745 (67.3%) passed at the first attempt. Excluding candidates with missing data, the largest groups within each variable were UKG (73.2%), female (51.2%), age >29 years old (53.7%), White (54.3%), no religion (35.6%), heterosexual/straight (96.1%), no disability (94.3%) and not LTFT (92.9%). The results of univariate analyses between sociodemographic variables and pass rates at all UK postgraduate medical examinations are shown in Table 1. Univariate analyses are not shown for each examination due to the size of the analyses output being too unwieldy for publication. However, these can be seen online using the GMC progression reports (https://edt.gmc-uk.org/progression-reports/specialty-examinations).

**Table 1.** Univariate analysis of postgraduate medical examination first attempt pass rates by sociodemographic variables for UK (UKG) and international medical graduates (IMG). Values presented as percentage pass rate and (number that passed/total number of first attempts (*N*)).

	Percentage pass rate at first attempt (number passed / total number of first attempts)		
	UK and international medical graduates	UK medical graduates	International medical graduates
***N* in cohort**	180,890	132,370	48,520
**PMQ** *p*-value	<0.001	N/A	N/A
UK	75.4% (99,840/132,370)	75.4% (99,840/132,370)	N/A
IMG	45.2% (21905/48515)	N/A	45.2% (21905/48515)
Missing (*N*)	0	0	0
**Gender** *p*-value	<0.001	<0.001	<0.001
Males	65.0% (57,415/88,280)	74.7% (44,970/60,165)	44.3% (12,450/28,115)
Females	69.5% (64,330/92,605)	76.0% (54,870/72,205)	46.4% (9460/20,400)
Missing (*N*)	0	0	0
**Age** *p*-value	<0.001	<0.001	<0.001
⩽29 years	71.9% (60,260/83,780)	74.6% (56,140/75,220)	48.2% (4120/8555)
>29 years	63.3% (61,485/97,110)	76.4% (43,700/57,150)	44.5% (17,785/39,960)
Missing (*N*)	0	0	0
**Ethnicity** *p*-value	<0.001	<0.001	<0.001
White	77.0% (72,550/94,180)	79.5% (68,055/85,565)	52.2% (4495/8615)
Asian or Asian British	56.7% (31,490/55,530)	67.6% (20,275/29,995)	43.9% (11,215/25,535)
Black or Black British	46.9% (4340/9250)	60.1% (1790/2980)	40.7% (2550/6270)
Mixed	68.2% (4055/5945)	74.2% (3575/4815)	42.5% (480/1130)
Other ethnic groups	55.3% (4755/8600)	65.1% (2655/4075)	46.4% (2100/4525)
Missing (*N*)	7385	4940	2445
**Religion** *p*-value	<0.001	<0.001	<0.001
No religion	77.5% (40,235/51,895)	79.0% (38,485/48,740)	55.5% (1750/3155)
Buddhist	57.9% (2145/3700)	64.7% (1235/1910)	50.7% (905/1785)
Christian	68.4% (32,015/46,805)	75.7% (27,075/35,760)	44.7% (4940/11045)
Hindu	56.5% (7995/14160)	69.2% (4370/6310)	46.2% (3625/7850)
Jewish	74.1% (725/975)	76.0% (675/890)	54.1% (45/85)
Muslim	50.5% (12,375/24,525)	64.6% (5425/8395)	43.1% (6950/16130)
Sikh	64.9% (1305/2015)	70.1% (1145/1630)	42.7% (165/380)
Other	63.2% (1150/1820)	69.1% (985/1425)	41.8% (165/395)
Missing (*N*)	35000	27310	7690
**Sexual orientation** *p*-value	<0.001	0.001	0.001
Heterosexual/Straight	67.0% (92,265/137,735)	75.6% (74,410/98,405)	45.4% (17,855/39,335)
Bisexual	67.5% (880/1305)	69.2% (770/1045)	41.3% (105/260)
Lesbian/Gay/Homosexual	72.2% (2755/3815)	74.0% (2550/3440)	55.1% (205/370)
Other	65.9% (330/500)	68.9% (285/410)	52.3% (45/90)
Missing (*N*)	37535	29070	8465
**Disability** *p*-value	<0.001	<0.001	<0.001
No vs	70.3% (99,475/14,1485)	76.4% (85,395/11,1715)	47.3% (14,080/29,770)
Yes	62.0% (5270/8500)	65.5% (4925/7520)	35.2% (345/980)
Missing (*N*)	30905	13135	17765
**LTFT** *p*-value	<0.001	<0.001	<0.001
No	66.9% (112,515/168,060)	75.0% (92,075/122,740)	45.1% (20,440/45,325)
Yes	72.0% (9230/12,830)	80.6% (7765/9635)	45.9% (1465/3195)
Missing (*N*)	0	0	0
**Prior attainment (*z* score)** *p*-value		<0.001	<0.001
Pass Mean	0.1210	0.0656	0.5599
Fail Mean	−0.0542	−0.1194	0.0674
Missing (*N*)	22500	3245	19260

**Figure 1.**
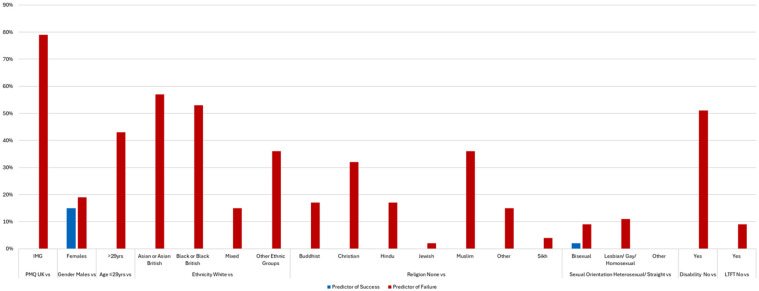
The percentage of UK postgraduate examinations where statistically significant differences in attainment have been found for UK (UKG) and international medical graduates (IMG) in regression analyses for each sociodemographic group.

The LR model heatmap showing independent predictors of success and failure at the first attempt at each postgraduate medical examination for all candidates after accounting for prior academic performance is shown in Supplemental Table 2. Full numerical LR results allowing for more granular analyses can be found in Supplemental Table 3. The percentage of UK postgraduate examinations where statistically significant differences in attainment have been found in regression analyses for each sociodemographic group is illustrated in Figure 1. Place of PMQ was found to be a strong independent predictor of failure. Compared with UKGs, IMGs were significantly less likely to pass 79% of examinations at the first attempt, irrespective of examination format (written or clinical). A notable exception was the First and Final FRCR Clinical Radiology examination outcomes, which were not predicted by PMQ after adjusting for other sociodemographic factors and prior academic attainment.

Gender-related DA was found across numerous examinations. Females were more likely to pass 15% of examinations (predominantly clinical), while males were more likely to pass 19% of examinations (predominantly written). A notable exception was the finding that females were more likely to pass the written and clinical components of the MRCGP (OR 1.13 (95% CI 1.03–1.25) and OR 2.18 (95% CI 1.94–2.44), respectively), while females were significantly less likely to pass both the written and clinical components of the MRCS examination (OR 0.55 (95% CI 0.49–0.62) and OR 0.77 (95% CI 0.65–0.92), respectively).

Older candidates (>29 years of age) were significantly less likely to pass 43% of the examinations. Drilling down, it seems that older age was a predictor of failure at more examinations commonly taken earlier in the training pathway (e.g. Royal College membership examinations, see earlier). Therefore, results for age must be interpreted at the examination level, considering when the examination is usually attempted in the training pathway (i.e. early or late). However, age-related DA was not found in most examinations.

Ethnicity was a strong predictor of outcomes at the first attempt at many examinations, with minority ethnic groups experiencing significantly lower pass rates than White candidates. Asian or Asian British and Black or Black British candidates were significantly less likely to pass at more than half of UK postgraduate medical examinations after adjusting for other sociodemographic factors and prior attainment. In some examinations, all minority ethnic groups (Asian or Asian British, Black or Black British, Mixed and other (non-White British) Ethnic groups) were significantly less likely to pass compared with White candidates (e.g. MRCGP, MRCP, MRCS and the Final FRCR Clinical Radiology written examinations). However, it is worth noting that the larger the cohort size, the greater the statistical power enabling regression models to highlight DA, and these examinations are several of the largest cohorts in the dataset. DA was present in the clinical but not the written components of some examinations, such as the FRCA Primary and MRCPsych.

There was a strong correlation (Spearman’s Rho) between ethnicity and religion *r* = 0.506 (*p* < 0.001), which is shown in Supplemental Table 4. However, even after adjusting for prior attainment and other sociodemographic factors that include ethnicity, religion-related DA still existed in 2–36% of examinations. DA was present according to religious beliefs in the written, but not the clinical components of some examinations (e.g. Final FRCA, FRCS, MRCOG, MRCP and MRCPsych).

Obvious DA was seen according to sexual orientation in several examinations. Bisexual candidates were significantly less likely than Straight or Heterosexual candidates to pass 9% of examinations while Lesbian, Gay or Homosexual candidates were less likely to pass 11% of examinations.

Candidates with registered disabilities were significantly less likely to pass at their first attempt in half of all examinations. DA, according to disability status, can be seen in both written and clinical components of some examinations, such as the MRCPsych, FRCA, FRCPath, MRCGP, MRCP and MRCPCH. LTFT status was found to be a statistically significant independent predictor of failure at four examinations: the Primary and Final FRCA and Final FRCR Clinical Oncology and Clinical Radiology clinical examinations.

Lastly, prior academic attainment remained a strong predictor of future outcomes in most postgraduate examinations.

## Discussion

These data highlight numerous sociodemographic factors that are statistically significant independent predictors of success or failure at UK postgraduate medical examinations.

In summary, after adjusting for numerous sociodemographic factors and prior academic attainment, significant differences in attainment were found according to place of PMQ, gender, age, ethnicity, religion, sexual orientation, disability and LTFT status. The strongest independent predictors of failing written and clinical examinations were being an international medical graduate, being from a minority ethnic group and having a registered disability.

Graduating from a medical school outside the UK was independently associated with significantly lower pass rates on UK postgraduate medical examinations. These findings confirm those of previous studies that found DA according to place of qualification in various individual UK postgraduate examinations.^19,20,22,23,25,29^ Thus, while IMGs make up more than half of registered doctors currently working in the UK,^38^ they are at the greatest risk of experiencing challenges in career progression where this is dependent on examination performance. Interestingly, other research also tells us that IMGs also experience significant differences in ‘on the job’ training outcomes (assessed via an Annual Review of Competency Progression (ARCP)) and recruitment into specialty training.^6,10,42^ This indicates a broader issue than examination-specific factors.

There is extensive literature on the challenges and barriers IMG doctors face both at the point of joining the UK workforce and thereafter. These are related to differences in language, culture and previous experiences of medical education and associated assessments, as well as a sense of a belonging in their new host country.^43^ There is also evidence that IMGs struggle with the educational structures of medicine and gaining social capital within the workplace.^44,45^ Many of these barriers are unique to IMGs, highlighting that some of the reasons for group-level differences may be related to specific demographics, while others may apply to both IMGs and UKs. It is also important to consider the intersectionality of identities, which will be discussed later.

This study found different patterns of attainment according to gender depending on examination format, with females being significantly less likely to pass some examinations (predominantly written), but significantly more likely to pass other examinations (predominantly clinical) at the first attempt than men. This pattern of DA is similar to that found in previous, single-specialty studies.^16,19,20,46–48^ The data in respect of gender were interesting, given that prior research suggests females might be expected to perform better at all types of examinations, given their higher attainment in medical school selection tests and during medical school.^49,50^ These gender differences require further exploration.

The analyses identified ethnicity as a strong independent predictor of postgraduate outcomes. Candidates from minority ethnic backgrounds, whether UK or overseas graduates, were significantly less likely to pass written and clinical examinations after adjusting for other sociodemographic factors. This finding is not unexpected. DA by ethnicity is a common pattern running through all levels of medical education and training. Medical students from minority ethnicity groups are significantly more likely to fail an assessment than their non-minority group peers^33,49^ and ethnicity-related DA has been found in many other individual studies of postgraduate assessment outcomes.^10,16–18,28,51^ In short, there are significant differences in attainment for candidates from minority ethnic groups compared with White candidates in UK medical postgraduate assessments and other progression hurdles.

To the best of our knowledge, this is the first study to look at the influence of religious beliefs on performance at most UK postgraduate medical exams adjusted for measures of prior attainment. After adjusting for all other sociodemographic variables, including ethnicity, candidates with declared religious beliefs were found to be significantly more likely to fail some written and some clinical examinations at the first attempt. These findings coupled with data on annual reviews of competence progression^10^ highlight the importance of including this factor when considering the intersectionality of different characteristics in relation to assessment outcomes and career progression. Qualitative studies suggest that religious beliefs may be associated with different experiences in medical training environments,^52–56^ suggesting the need for larger-scale, conceptually-framed national studies to comprehensively explore differences in experiences between different religious groups in the training, assessment and workplace environments.

We also found that DA, according to religious beliefs, is predominantly seen in the written components and not the clinical components of some examinations. This suggests the need to examine bias, unfairness and discrimination at the written question level (see later for further discussion).

There were statistically significant differences in attainment according to sexual orientation. Sexual orientation has historically been neglected by previous, similar data collection exercises, and limited categorisation exists in the current database for analysis. Additionally, analyses can be challenging due to small numbers, which may not be entirely representative given that more than half of LGBTQ+ doctors do not feel comfortable being open with their sexual orientation and/or gender identity.^57^ However, these data still provide useful information that LGBTQ+ doctors experience differences in attainment compared with their peers. That DA exists according to sexual orientation is not unexpected, given the myriad articles in the literature that describe the lack of mentorship and role models, frequent bias, minoritisation, discrimination and harassment that LGBTQ+ doctors experience within the workplace and training environments.^57,58^ These and the current study indicate the need to further explore differences in experiences, opportunities and attainment due to sexual orientation and gender identity.

Disability was a strong independent predictor of failure in around half of all UK postgraduate medical examinations. This is an important new finding given that GMC data suggest lower reporting of disability by IMGs and minoritised groups, which has potentially hidden this finding from previous research that used only descriptive and univariate analyses. As with all the DA identified, it is critical to investigate factors impacting the performance of doctors with disabilities. These may lie in the assessment environment or be due to differences in experiences and opportunities in the learning and training environment, or both. Medical Royal Colleges and Postgraduate Training Deaneries should also investigate the effectiveness of current reasonable adjustments (referring to removing barriers or providing support, such as extra time in examinations) to enable performance unrestricted by disability.

LTFT was found to be an independent predictor of failing four examinations. LTFT doctors are a heterogeneous cohort. Doctors choose LTFT for a broad range of reasons, which may include childcare, caring responsibilities, health and wellbeing, disability or ill-health, prevention of burnout, work–life balance, hobbies, academic and business interests, studying and opportunities for personal or professional development, to name but a few. LTFT training rates also differ between specialties, years in training, ethnicity and gender.^38^ LTFT trainees also differ in terms of their experiences within the workplace and training environment. This group merits more attention, including a more granular look at the impact of LTFT on postgraduate assessment outcomes (and reasons for choosing to work LTFT) and in-depth qualitative work to explore corresponding workplace experiences. A greater understanding of this interplay would help to guide efforts to reduce its impact on postgraduate examination performance and career progression. This is especially important given the number of doctors choosing to work less than full-time has more than doubled in the last 10 years to 18%. Therefore, DA in career progression is likely to have a considerable impact on a large number of doctors, influencing workforce planning.

### Future work

Although our focus is postgraduate examinations, the four broad areas with explanatory potential proposed by Mountford-Zimdars et al.’s 2015 review of DA in undergraduate studies across the UK are useful areas to consider for future research.^59^ These are learners’ experience of the curriculum (including teaching and assessment practices, not just formal examinations and assessments), psychosocial and identity factors (including internal and external expectations on learners), and their social and cultural capital (including relationships between trainers and learners, between learners and an overall sense of belonging and support). These areas are also reflected in Fyfe et al.’s 2021 review that proposed how to redress DA related to ethnicity in medical schools.^5^ In short, it is necessary to look at the entire system (including differences in the delivery and structure of specialty training structures and workplace cultures) to root out inequity of opportunities and experiences preventing one group of candidates from progressing in their specialty training at the same rate as the other, and to guide future interventions aiming to reduce these attainment gaps. This research should be undertaken with consideration of the intersectionality of identities and the knowledge that some groups will be experiencing the accumulation of sociocultural and economic disadvantages (‘double jeopardy’).

At an examination level, these data highlighting differences in attainment between groups can be used by Medical Royal Colleges, the GMC and postgraduate training organisations or directors of medical education to guide more granular research at the specialty level (looking at access to local training opportunities within each specialty, for example), and at an examination and question level, including test format, differential-item functioning and linguistic analyses to ensure examination bias and discrimination can be ruled out as a causal factor for DA. These data will also guide interventions and change efforts aiming to reduce this awarding gap in the postgraduate training environment.^11,12^

### Strengths and limitations

This, to date, was the largest study of outcomes across all UK postgraduate medical examinations and one of the few studies in this area that account for prior academic attainment, has highlighted sociodemographic differences that are independently predictive of success or failure in both the written and clinical components of each examination. This study progresses the existing understanding of which factors have an impact on later postgraduate outcomes; the predictive factors identified in this study include several that have received much attention in the literature but also include others that have received less attention in data collection exercises and DA studies. This study highlights the importance of considering other factors such as these, which, include sociodemographic differences not listed as protected characteristics, in data collection exercises and future research.

As is common to all retrospective studies, analyses are limited by the data that has been collected historically. All sociodemographic differences collected in the GMC database were used in MV models, but there are inevitably unknown unknowns that do not yet exist in the database; confounding factors that may impact performance on postgraduate examinations. As data collection evolves, these variables may become available for analysis and may shed more light on the variance behind attainment in examinations. In addition to the sociodemographic factors analysed in this study, GMC holds data linked on an individual-level for multiple measures of socioeconomic status and educational background; factors that have been shown to impact outcomes on some postgraduate assessments.^18^ However, these data are collected at the point of application to medical school for UKGs and there is no equivalent point of data collection for IMGs. Given that fewer than 5% of IMGs had matched data for these measures, we made the decision not to carry these forward into the regression analyses. A study is underway to explore whether these factors are also independent predictors of success or failure in postgraduate medical examinations, and whether attainment patterns differ when UKGs are analysed independently of IMGs.

Throughout the study, variables were dichotomised or categorised (see Methods). While this approach is pragmatic and commonly used when studying group differences,^10^ it does limit granularity when considering the intersectionality of identities.^5,30,60^ Furthermore, group aggregation hides the heterogeneity within each group. For example, IMGs in the UK are from many different countries across the world.^38^ They differ in terms of language, culture and heritage and so are likely to have had considerable inter and intra-group differences in social and educational opportunities and lived experiences that are not represented within these data. Similarly, how social identities and groups are categorised is fluid and frequently changing. These changes are rarely represented within quantitative datasets.

The current MV models are presented as complete case analyses; candidates with missing data for one or more of the predictors of success are excluded from the final model. We cannot, therefore, exclude the possibility of bias in that those candidates excluded may have had a different demographic profile and/or success rate when compared with the included candidates. On the other hand, a few candidates may have attempted multiple examinations and may be represented more than once in the data.

We acknowledge that multiple statistical comparisons have been performed, although use of a simple Bonferroni adjustment would, in our opinion, be a conservative strategy. Therefore, we suggest caution in interpreting effect sizes that are around the conventional <0.05 statistical significance; perhaps focusing more on those around the <0.01 significance level. Confidence intervals should also be examined to reflect the precision of the estimated effect size.

## Conclusions

This was the largest study to date investigating independent predictors of outcomes at UK postgraduate medical examinations, adjusted for prior academic attainment. Place of primary qualification, gender, age, ethnicity, religion, sexual orientation, disability and LTFT status were all found to be statistically significant independent predictors of success or failure in written and clinical examinations. These data can be used by Medical Royal Colleges, the GMC, Deaneries and training institutions to guide more granular research and future change efforts, aiming to reduce the attainment gap highlighted by this study.

## Supplemental Material

sj-docx-1-jrs-10.1177_01410768251380980. – Supplemental material for A multivariate analysis to identify the relationship between sociodemographic differences and examination performance in UK postgraduate medical examinations Supplemental material, sj-docx-1-jrs-10.1177_01410768251380980. for A multivariate analysis to identify the relationship between sociodemographic differences and examination performance in UK postgraduate medical examinations by Ricky Ellis, Andy Knapton, Jane Cannon, Amanda J Lee and Jennifer Cleland in Journal of the Royal Society of Medicine

sj-docx-2-jrs-10.1177_01410768251380980. – Supplemental material for A multivariate analysis to identify the relationship between sociodemographic differences and examination performance in UK postgraduate medical examinations Supplemental material, sj-docx-2-jrs-10.1177_01410768251380980. for A multivariate analysis to identify the relationship between sociodemographic differences and examination performance in UK postgraduate medical examinations by Ricky Ellis, Andy Knapton, Jane Cannon, Amanda J Lee and Jennifer Cleland in Journal of the Royal Society of Medicine

sj-docx-3-jrs-10.1177_01410768251380980. – Supplemental material for A multivariate analysis to identify the relationship between sociodemographic differences and examination performance in UK postgraduate medical examinations Supplemental material, sj-docx-3-jrs-10.1177_01410768251380980. for A multivariate analysis to identify the relationship between sociodemographic differences and examination performance in UK postgraduate medical examinations by Ricky Ellis, Andy Knapton, Jane Cannon, Amanda J Lee and Jennifer Cleland in Journal of the Royal Society of Medicine

sj-docx-4-jrs-10.1177_01410768251380980. – Supplemental material for A multivariate analysis to identify the relationship between sociodemographic differences and examination performance in UK postgraduate medical examinations Supplemental material, sj-docx-4-jrs-10.1177_01410768251380980. for A multivariate analysis to identify the relationship between sociodemographic differences and examination performance in UK postgraduate medical examinations by Ricky Ellis, Andy Knapton, Jane Cannon, Amanda J Lee and Jennifer Cleland in Journal of the Royal Society of Medicine

## Declarations

**Data availability statement:** The dataset used in this study is collected by the GMC as part of its statutory duty to assure the quality of medical education and training. Data were deidentified by GMC staff before being provided to the researchers for analysis and interpretation of findings. Due to data protection regulations, the source data cannot be supplied. Anonymized summaries of it are supplied in this manuscript.

**Ethics approval:** No formal ethical approval was required for this study of existing, anonymised GMC data.

**Guarantor:** RE.

**Provenance:** Not commissioned; peer-reviewed by Olwyn Margaret Westwood and Julie Morris.

**Supplemental material:** Supplemental material for this article is available online.

**ORCID iD:** Ricky Ellis  https://orcid.org/0000-0001-7310-6904
