# Supplementary material for: A multivariate analysis to identify the relationship between sociodemographic differences and examination performance in UK postgraduate medical examinations
Source: J R Soc Med. 2025 Nov 3;118(10):325–35. doi: 10.1177/01410768251380980 (PMC12583007; doi:10.1177/01410768251380980)
Supplement: sj-docx-1-jrs-10.1177_01410768251380980. – Supplemental material for A multivariate analysis to identify the relationship between sociodemographic differences and examination performance in UK postgraduate medical examinations [file sj-docx-1-jrs-10.1177_01410768251380980.docx]

**Supplementary Table 1.** Examination key. Examination titles as recorded in the UKMED database and the abbreviations used in this study.

| **Examination title** | **Abbreviation** |
| --- | --- |
| Diploma in Otolaryngology – Head and Neck Surgery OSCE | DOHNS Clinical |
| Fellowship of the Faculty of Intensive Care Medicine MCQ | FFICM Written |
| Fellowship of the Faculty of Intensive Care Medicine OSCE & Structured Oral Examination (SOE) | FFICM Clinical |
| Final Fellowship of The Royal College of Radiologists (Part A) Clinical Oncology | Fin FRCR CO-A Written |
| Final Fellowship of The Royal College of Radiologists (Part A) Clinical Radiology | Fin FRCR CR-A Written |
| Final Fellowship of The Royal College of Radiologists (Part B) Clinical Oncology | Fin FRCR CO-B Clin |
| Final Fellowship of The Royal College of Radiologists (Part B) Clinical Radiology | Fin FRCR CR-B Clin |
| First Fellowship of The Royal College of Radiologists Clinical Oncology | Fir FRCR CO Written |
| First Fellowship of The Royal College of Radiologists Clinical Radiology | Fir FRCR CR Written |
| Fellowship of the Royal College of Anaesthetists Final SOE | FRCA Fin Clinical |
| Fellowship of the Royal College of Anaesthetists Final Written | FRCA Fin Written |
| Fellowship of the Royal College of Anaesthetists Primary MCQ | FRCA Pri Written |
| Fellowship of the Royal College of Anaesthetists Primary OSCE & SOE | FRCA Pri Clinical |
| Fellowship of the Royal College of Emergency Medicine OSCE | FRCEM Clinical |
| Fellowship of the Royal College of Emergency Medicine Written Components | FRCEM Written |
| Fellowship Exam of the Royal College of Ophthalmologists Part 1 | FRCOphth 1 Written |
| Fellowship Exam of the Royal College of Ophthalmologists Part 2 Oral | FRCOphth 2 Clinical |
| Fellowship Exam of the Royal College of Ophthalmologists Part 2 Written | FRCOphth 2 Written |
| Fellowship of the Royal College of Pathologists Part 1 | FRCPath 1 Written |
| Fellowship of the Royal College of Pathologists Part 2 | FRCPath 2 |
| Fellowship of the Royal Colleges of Surgeons Part 1 | FRCS Written |
| Fellowship of the Royal Colleges of Surgeons Part 2 | FRCS Clinical |
| Membership of Royal College of Emergency Medicine OSCE | MRCEM Clinical |
| Membership of Royal College of Emergency Medicine Written Components | MRCEM Written |
| Membership of Royal College of General Practice Applied Knowledge Test | MRCGP Written |
| Membership of Royal College of General Practice Clinical Skills Assessment | MRCGP Clinical |
| Membership of Royal College of Obstetrics & Gynaecology Part 3 | MRCOG Clinical |
| Membership of Royal College of Obstetrics & Gynaecology Parts 1 and 2 | MRCOG Written |
| Membership of the Royal College of Physicians Paces | MRCP Clinical |
| Membership of the Royal College of Physicians Part 1 | MRCP 1 Written |
| Membership of the Royal College of Physicians Part 2 | MRCP 2 Written |
| Membership of the Royal College of Paediatrics and Child Health | MRCPCH Written |
| Membership of the Royal College of Paediatrics and Child Health Clinical | MRCPCH Clinical |
| Membership of the Royal Colleges of Surgeons Part A | MRCS Written |
| Membership of the Royal Colleges of Surgeons Part B | MRCS Clinical |
| Royal College of Ophthalmologists Refraction Certificate (Clinical) | RCOphth Ref Cert |
| Membership of the Royal College of Psychiatrists Clinical Assessment of Skills and Competencies | MRCPsych Clinical |
| Membership of the Royal College of Psychiatrists Paper A | MRCPsych A Written |
| Membership of the Royal College of Psychiatrists Paper B | MRCPsych B Written |
| Specialty Certificate Examination (SCE) Acute Medicine (Written) | Acute Med |
| SCE Dermatology (Written) | Dermatology |
| SCE Endocrinology and Diabetes (Written) | Endo & Diabetes |
| SCE Gastroenterology (Written) | Gastroenterology |
| SCE Geriatric Medicine (Written) | Geriatric Med |
| SCE Nephrology (Written) | Nephrology |
| SCE Palliative Medicine (Written) | Palliative Med |
| SCE Respiratory Medicine (Written) | Respiratory Med |
