# Supplementary material for: A multivariate analysis to identify the relationship between sociodemographic differences and examination performance in UK postgraduate medical examinations
Source: J R Soc Med. 2025 Nov 3;118(10):325–35. doi: 10.1177/01410768251380980 (PMC12583007; doi:10.1177/01410768251380980)
Supplement: sj-docx-2-jrs-10.1177_01410768251380980. – Supplemental material for A multivariate analysis to identify the relationship between sociodemographic differences and examination performance in UK postgraduate medical examinations [file sj-docx-2-jrs-10.1177_01410768251380980.docx]

**Supplementary Table 2.** Logistic regression model heatmap (and odds ratio key) showing predictors of success at the first attempt at each postgraduate medical examination for UK (UKG) and International Medical graduates (IMG) after accounting for prior academic performance. The first category displayed within each variable was used as the reference. Examination abbreviations are outlined in the examination key.

|  | DOHNS Clinical | FFICM Written | FFICM Clinical | Fin FRCR CO-A Written | Fin FRCR CR-A Written | Fin FRCR CO-B Clin | Fin FRCR CR-B Clin | Fir FRCR CO Written | Fir FRCR CR Written | FRCA Fin Clinical | FRCA Fin Written | FRCA Pri Written | FRCA Pri Clinical | FRCEM Clinical | FRCEM Written | FRC Ophth 1 Written | FRC Ophth 2 Clinical | FRC Ophth 2 Written | FRCPath 1 Written | FRCPath 2 | FRCS Written | FRCS Clinical | MRCEM Clinical | MRCEM Written | MRCGP Written | MRCGP Clinical | MRCOG Clinical | MRCOG Written | MRCP Clinical | MRCP 1 Written | MRCP 2 Written | MRCPCH Written | MRCPCH Clinical | MRCS Written | MRCS Clinical | RCOphth Ref Cert | MRCPsych Clinical | MRCPsych A Written | MRCPsych B Written | Acute Med | Dermatology | Endo & Diabetes | Gastroenterology | Geriatric Med | Nephrology | Palliative Med | Respiratory Med |
| --- | --- | --- | --- | --- | --- | --- | --- | --- | --- | --- | --- | --- | --- | --- | --- | --- | --- | --- | --- | --- | --- | --- | --- | --- | --- | --- | --- | --- | --- | --- | --- | --- | --- | --- | --- | --- | --- | --- | --- | --- | --- | --- | --- | --- | --- | --- | --- |
| **N in analysis** | 365 | 535 | 450 | 240 | 1070 | 220 | 935 | 330 | 1035 | 2135 | 2235 | 2850 | 2820 | 520 | 1890 | 550 | 350 | 410 | 760 | 660 | 2440 | 2180 | 415 | 2755 | 13525 | 11935 | 585 | 2275 | 7350 | 12820 | 8170 | 3535 | 2200 | 5200 | 3005 | 370 | 1845 | 1635 | 1450 | 385 | 250 | 310 | 265 | 685 | 225 | 220 | 505 |
| **PMQ**  UK vs | | | | | | | | | | | | | | | | | | | | | | | | | | | | | | | | | | | | | | | | | | | | | | | |
| IMG |  |  |  |  |  |  |  |  |  |  |  |  |  |  |  |  |  |  |  |  |  |  |  |  |  |  |  |  |  |  |  |  |  |  |  |  |  |  |  |  |  |  |  |  |  |  |  |
| **Gender** Males vs | | | | | | | | | | | | | | | | | | | | | | | | | | | | | | | | | | | | | | | | | | | | | | | |
| Females |  |  |  |  |  |  |  |  |  |  |  |  |  |  |  |  |  |  |  |  |  |  |  |  |  |  |  |  |  |  |  |  |  |  |  |  |  |  |  |  |  |  |  |  |  |  |  |
| **Age** ≤29yrs vs | | | | | | | | | | | | | | | | | | | | | | | | | | | | | | | | | | | | | | | | | | | | | | | |
| >29yrs |  |  |  |  |  |  |  |  |  |  |  |  |  |  |  |  |  |  |  |  |  |  |  |  |  |  |  |  |  |  |  |  |  |  |  |  |  |  |  |  |  |  |  |  |  |  |  |
| **Ethnicity** White vs | | | | | | | | | | | | | | | | | | | | | | | | | | | | | | | | | | | | | | | | | | | | | | | |
| Asian or Asian British |  |  |  |  |  |  |  |  |  |  |  |  |  |  |  |  |  |  |  |  |  |  |  |  |  |  |  |  |  |  |  |  |  |  |  |  |  |  |  |  |  |  |  |  |  |  |  |
| Black or Black British |  |  |  |  |  |  |  |  |  |  |  |  |  |  |  |  |  |  |  |  |  |  |  |  |  |  |  |  |  |  |  |  |  |  |  |  |  |  |  |  |  |  |  |  |  |  |  |
| Mixed |  |  |  |  |  |  |  |  |  |  |  |  |  |  |  |  |  |  |  |  |  |  |  |  |  |  |  |  |  |  |  |  |  |  |  |  |  |  |  |  |  |  |  |  |  |  |  |
| Other Ethnic Groups |  |  |  |  |  |  |  |  |  |  |  |  |  |  |  |  |  |  |  |  |  |  |  |  |  |  |  |  |  |  |  |  |  |  |  |  |  |  |  |  |  |  |  |  |  |  |  |
| **Religion** None vs | | | | | | | | | | | | | | | | | | | | | | | | | | | | | | | | | | | | | | | | | | | | | | | |
| Buddhist |  |  |  |  |  |  |  |  |  |  |  |  |  |  |  |  |  |  |  |  |  |  |  |  |  |  |  |  |  |  |  |  |  |  |  |  |  |  |  |  |  |  |  |  |  |  |  |
| Christian |  |  |  |  |  |  |  |  |  |  |  |  |  |  |  |  |  |  |  |  |  |  |  |  |  |  |  |  |  |  |  |  |  |  |  |  |  |  |  |  |  |  |  |  |  |  |  |
| Hindu |  |  |  |  |  |  |  |  |  |  |  |  |  |  |  |  |  |  |  |  |  |  |  |  |  |  |  |  |  |  |  |  |  |  |  |  |  |  |  |  |  |  |  |  |  |  |  |
| Jewish |  |  |  |  |  |  |  |  |  |  |  |  |  |  |  |  |  |  |  |  |  |  |  |  |  |  |  |  |  |  |  |  |  |  |  |  |  |  |  |  |  |  |  |  |  |  |  |
| Muslim |  |  |  |  |  |  |  |  |  |  |  |  |  |  |  |  |  |  |  |  |  |  |  |  |  |  |  |  |  |  |  |  |  |  |  |  |  |  |  |  |  |  |  |  |  |  |  |
| Other |  |  |  |  |  |  |  |  |  |  |  |  |  |  |  |  |  |  |  |  |  |  |  |  |  |  |  |  |  |  |  |  |  |  |  |  |  |  |  |  |  |  |  |  |  |  |  |
| Sikh |  |  |  |  |  |  |  |  |  |  |  |  |  |  |  |  |  |  |  |  |  |  |  |  |  |  |  |  |  |  |  |  |  |  |  |  |  |  |  |  |  |  |  |  |  |  |  |
| **Sexual Orientation** Heterosexual/ Straight vs | | | | | | | | | | | | | | | | | | | | | | | | | | | | | | | | | | | | | | | | | | | | | | | |
| Bisexual |  |  |  |  |  |  |  |  |  |  |  |  |  |  |  |  |  |  |  |  |  |  |  |  |  |  |  |  |  |  |  |  |  |  |  |  |  |  |  |  |  |  |  |  |  |  |  |
| Lesbian/ Gay/Homosexual |  |  |  |  |  |  |  |  |  |  |  |  |  |  |  |  |  |  |  |  |  |  |  |  |  |  |  |  |  |  |  |  |  |  |  |  |  |  |  |  |  |  |  |  |  |  |  |
| Other |  |  |  |  |  |  |  |  |  |  |  |  |  |  |  |  |  |  |  |  |  |  |  |  |  |  |  |  |  |  |  |  |  |  |  |  |  |  |  |  |  |  |  |  |  |  |  |
| **Disability**  No vs | | | | | | | | | | | | | | | | | | | | | | | | | | | | | | | | | | | | | | | | | | | | | | | |
| Yes |  |  |  |  |  |  |  |  |  |  |  |  |  |  |  |  |  |  |  |  |  |  |  |  |  |  |  |  |  |  |  |  |  |  |  |  |  |  |  |  |  |  |  |  |  |  |  |
| **LTFT** No vs | | | | | | | | | | | | | | | | | | | | | | | | | | | | | | | | | | | | | | | | | | | | | | | |
| Yes |  |  |  |  |  |  |  |  |  |  |  |  |  |  |  |  |  |  |  |  |  |  |  |  |  |  |  |  |  |  |  |  |  |  |  |  |  |  |  |  |  |  |  |  |  |  |  |
|  | | | | | | | | | | | | | | | | | | | | | | | | | | | | | | | | | | | | | | | | | | | | | | | |
| **Prior Attainment** |  |  |  |  |  |  |  |  |  |  |  |  |  |  |  |  |  |  |  |  |  |  |  |  |  |  |  |  |  |  |  |  |  |  |  |  |  |  |  |  |  |  |  |  |  |  |  |

| **Odds ratio key** | | |
| --- | --- | --- |
| ≥1.51 |  | Strong predictor of doing better at the Examination |
| 1.26 – 1.50 |  |  |
| 1.01 - 1.25 |  |  |
| 1.00 or OR not statistically significant |  |  |
| 0.75 - 0.99 |  |  |
| 0.5 - 0.74 |  |  |
| ≤0.49 |  | Strong predictor of doing worse at the Examination |
| Not Applicable |  | Insufficient cohort size |
