# Supplementary material for: A multivariate analysis to identify the relationship between sociodemographic differences and examination performance in UK postgraduate medical examinations
Source: J R Soc Med. 2025 Nov 3;118(10):325–35. doi: 10.1177/01410768251380980 (PMC12583007; doi:10.1177/01410768251380980)
Supplement: sj-docx-3-jrs-10.1177_01410768251380980. – Supplemental material for A multivariate analysis to identify the relationship between sociodemographic differences and examination performance in UK postgraduate medical examinations [file sj-docx-3-jrs-10.1177_01410768251380980.docx]

**Supplementary Table 3.** Logistic regression model showing predictors of success at the first attempt at each postgraduate medical examination for UK (UKG) and International Medical graduates (IMG) after accounting for prior academic performance. The first category within each variable was used as the reference. Results are presented as odds ratio (95% confidence interval) and statistically significant effect sizes are boldened. Only variables reaching significance in the final model are displayed. ‘X’ denotes an invalid model due to small cohort sizes and ‘-‘ denotes where a variable has not met statistical significance within the final model. Examination abbreviations are outlined in the examination key.

| **A** | DOHNS Clinical | FFICM Written | FFICM Clinical | Fin FRCR CO-A Written | Fin FRCR CR-A Written | Fin FRCR CO-B Clin | Fin FRCR CR-B Clin | Fir FRCR CO Written | Fir FRCR CR Written | FRCA Fin Clinical | FRCA Fin Written | FRCA Pri Written | FRCA Pri Clinical |
| --- | --- | --- | --- | --- | --- | --- | --- | --- | --- | --- | --- | --- | --- |
| **N in analysis** | 365 | 535 | 450 | 240 | 1070 | 220 | 935 | 330 | 1035 | 2135 | 2235 | 2850 | 2820 |
| **PMQ**  UK vs |  |  |  |  |  |  |  |  |  |  |  |  |  |
| IMG | 0.34 | **0.23** | **0.25** | **0.27** | - | **0.28** | 0.69 | - | - | **0.45** | **0.37** | **0.54** | **0.43** |
|  | (0.11-1.03) | **(0.10-0.54)** | **(0.11-0.58)** | **(0.13-0.56)** | - | **(0.13-0.59)** | (0.44-1.07) | - | - | **(0.30-0.68)** | **(0.24-0.57)** | **(0.34-0.85)** | **(0.26-0.72)** |
| **Gender** Males vs |  |  |  |  |  |  |  |  |  |  |  |  |  |
| Females | - | **0.41** | - | - | - |  | **1.58** | - | 0.76 | - | - | **0.59** | **-** |
|  | - | **(0.24-0.69)** | - | - | - |  | **(1.12-2.22)** | - | (0.56-1.04) | - | - | **(0.51-0.70)** | **-** |
| **Age** ≤29yrs vs |  |  |  |  |  |  |  |  |  |  |  |  |  |
| >29yrs | **0.36** | - | - | - | - | X | **0.43** | **0.55** | - | **0.71** | **0.65** | 0.83 | **0.77** |
|  | **(0.21-0.64)** | - | - | - | - | X | **(0.28-0.66)** | **(0.33-0.93)** | - | **(0.53-0.95)** | **(0.51-0.83)** | (0.68-1.01) | **(0.64-0.92)** |
| **Ethnicity** White vs |  |  |  |  |  |  |  |  |  |  |  |  |  |
| Asian or Asian British | **0.44** | 0.56 | - | - | **0.64** | - | **0.44** | - | **0.60** | **0.46** | **0.44** | - | **0.59** |
|  | **(0.22-0.86)** | (0.29-1.10) | - | - | **(0.44-0.93)** | - | **(0.28-0.69)** | - | **(0.37-0.96)** | **(0.36-0.59)** | **(0.32-0.61)** | - | **(0.44-0.78)** |
| Black or Black British | 0.38 | **0.14** | - | - | **0.42** | - | 0.71 | - | **0.24** | 0.73 | **0.31** | - | **0.24** |
|  | (0.12-1.17) | **(0.02-0.92)** | - | - | **(0.20-0.88)** | - | (0.29-1.78) | - | **(0.12-0.47)** | (0.29-1.85) | **(0.14-0.70)** | - | **(0.08-0.67)** |
| Mixed | 1.97 | 0.35 | - | - | **0.49** | - | **0.39** | - | 1.37 | 0.71 | 0.65 | - | 0.74 |
|  | (0.23-16.80) | (0.11-1.11) | - | - | **(0.26-0.92)** | - | **(0.18-0.84)** | - | (0.46-4.07) | (0.43-1.16) | (0.40-1.04) | - | (0.50-1.10) |
| Other Ethnic Groups | 0.34 | 0.58 | - | - | **0.43** | - | 0.58 | - | 0.51 | **0.42** | 0.75 | - | **0.40** |
|  | (0.11-1.04) | (0.13-2.52) | - | - | **(0.23-0.79)** | - | (0.28-1.20) | - | (0.26-1.02) | **(0.23-0.77)** | (0.40-1.43) | - | **(0.23-0.72)** |
| **Religion** None vs |  |  |  |  |  |  |  |  |  |  |  |  |  |
| Buddhist | 0.61 | - | - | - | 0.66 | - | 0.57 | 1.36 | 1.53 | - | 0.78 | 0.74 | **0.49** |
|  | (0.13-2.82) | - | - | - | (0.26-1.67) | - | (0.22-1.50) | (0.11-17.29) | (0.41-5.71) | - | (0.35-1.73) | (0.38-1.43) | **(0.25-0.98)** |
| Christian | 0.65 | - | - | - | 0.78 | - | **0.60** | 0.70 | 0.68 | - | **0.75** | **0.71** | 0.87 |
|  | (0.36-1.19) | - | - | - | (0.57-1.08) | - | **(0.39-0.90)** | (0.41-1.20) | (0.44-1.05) | - | **(0.61-0.93)** | **(0.60-0.85)** | (0.73-1.04) |
| Hindu | 0.71 | - | - | - | **0.55** | - | 1.09 | 0.60 | **0.43** | - | 0.77 | **0.55** | 0.99 |
|  | (0.27-1.83) | - | - | - | **(0.33-0.89)** | - | (0.60-1.98) | (0.28-1.29) | **(0.24-0.79)** | - | (0.48-1.23) | **(0.39-0.78)** | (0.65-1.50) |
| Jewish | X | - | - | - | X | - | 0.25 | 0.45 | 0.56 | - | 0.42 | 1.08 | 1.07 |
|  | X | - | - | - | X | - | (0.05-1.36) | (0.04-5.29) | (0.03-4.39) | - | (0.16-1.10) | (0.33-3.52) | (0.38-2.99) |
| Muslim | 1.69 | - | - | - | **0.47** | - | 0.72 | **0.26** | **0.54** | - | **0.61** | **0.44** | **0.57** |
|  | (0.61-4.68) | - | - | - | **(0.31-0.72)** | - | (0.42-1.21) | **(0.10-0.67)** | **(0.32-0.91)** | - | **(0.38-0.98)** | **(0.30-0.65)** | **(0.36-0.89)** |
| Other | 1.58 | - | - | - | 1.22 | - | 2.30 | X | **0.24** | - | 0.49 | 0.62 | 0.93 |
|  | (0.10-25.67) | - | - | - | (0.34-4.40) | - | (0.26-20.62) | X | **(0.07-0.89)** | - | (0.19-1.23) | (0.29-1.34) | (0.42-2.08) |
| Sikh | X | - | - | - | 0.91 | - | 1.24 | X | 0.39 | - | 0.43 | 0.55 | 0.62 |
|  | X | - | - | - | (0.39-2.13) | - | (0.46-3.35) | X | (0.14-1.13) | - | (0.17-1.07) | (0.28-1.08) | (0.30-1.27) |
| **Sexual Orientation** Heterosexual/ Straight vs |  |  |  |  |  |  |  |  |  |  |  |  |  |
| Bisexual | - | **0.11** | - | - | - | - | - | **3.48** | - | 0.40 | 0.82 | - | - |
|  | - | **(0.02-0.54)** | - | - | - | - | - | **(1.03-11.79)** | - | (0.13-1.17) | (0.27-2.45) | - | - |
| Lesbian/ Gay/ Homosexual | - | 0.43 | - | - | - | - | - | X | - | 0.77 | **0.49** | - | - |
|  | - | (0.15-1.23) | - | - | - | - | - | X | - | (0.46-1.28) | **(0.31-0.79)** | - | - |
| Other | - | - | - | - | - | - | - | - | - | X | 0.46 | - | - |
|  | - | - | - | - | - | - | - | - | - | X | (0.11-1.87) | - | - |
| **Disability**  No vs |  |  |  |  |  |  |  |  |  |  |  |  |  |
| Yes | - | - | - | - | **0.54** | - | **0.46** | - | **0.54** | **0.61** | **0.50** | **0.55** | **0.59** |
|  | - | - | - | - | **(0.30-0.94)** | - | **(0.23-0.91)** | - | **(0.30-0.98)** | **(0.38-0.97)** | **(0.32-0.76)** | **(0.40-0.76)** | **(0.42-0.82)** |
| **LTFT** No vs |  |  |  |  |  |  |  |  |  |  |  |  |  |
| Yes | - | - | - | - | - | **0.49** | **0.55** | - | - | **0.69** | - | - | **0.53** |
|  | - | - | - | - | - | **(0.24-0.99)** | **(0.34-0.89)** | - | - | **(0.50-0.95)** | - | - | **(0.34-0.84)** |
| **Prior Attainment** | - | - | - | - | **1.28** | 1.30 | - | **1.28** | **1.32** | **1.18** | **1.20** | **1.24** | **1.24** |
|  | - | - | - | - | **(1.12-1.45)** | (0.96-1.78) | - | **(1.00-1.65)** | **(1.13-1.55)** | **(1.07-1.30)** | **(1.09-1.32)** | **(1.13-1.35)** | **(1.14-1.35)** |

| **B** | FRCEM Clinical | FRCEM Written | FRC Ophth 1 Written | FRC Ophth 2 Clinical | FRC Ophth 2 Written | FRCPath 1 Written | FRCPath 2 | FRCS Written | FRCS Clinical | MRCEM Clinical | MRCEM Written | MRCGP Written | MRCGP Clinical |
| --- | --- | --- | --- | --- | --- | --- | --- | --- | --- | --- | --- | --- | --- |
| **N in analysis** | 520 | 1890 | 550 | 350 | 410 | 760 | 660 | 2440 | 2180 | 415 | 2755 | 13525 | 11935 |
| **PMQ**  UK vs |  |  |  |  |  |  |  |  |  |  |  |  |  |
| IMG | **0.19** | **0.25** | - | **0.36** | - | **0.53** | **0.28** | **0.33** | **0.23** | **0.07** | **0.31** | **0.26** | **0.15** |
|  | **(0.05-0.63)** | **(0.18-0.36)** | - | **(0.18-0.72)** | - | **(0.30-0.92)** | **(0.18-0.43)** | **(0.24-0.44)** | **(0.17-0.32)** | **(0.01-0.37)** | **(0.23-0.41)** | **(0.22-0.29)** | **(0.13-0.18)** |
| **Gender** Males vs |  |  |  |  |  |  |  |  |  |  |  |  |  |
| Females | - | **-** | **0.63** | - | - | - | - | **0.53** | **-** | - | **0.75** | **1.13** | **2.18** |
|  | - | **-** | **(0.44-0.89)** | - | - | - | - | **(0.42-0.69)** | **-** | - | **(0.62-0.89)** | **(1.03-1.25)** | **(1.94-2.44)** |
| **Age** ≤29yrs vs |  |  |  |  |  |  |  |  |  |  |  |  |  |
| >29yrs | - | **0.68** | **-** | - | **0.47** | - | - | **-** | **-** | - | **-** | **0.77** | **0.65** |
|  | - | **(0.52-0.90)** | **-** | - | **(0.22-0.98)** | - | - | **-** | **-** | - | **-** | **(0.68-0.86)** | **(0.55-0.76)** |
| **Ethnicity** White vs |  |  |  |  |  |  |  |  |  |  |  |  |  |
| Asian or Asian British | 0.61 | **0.29** | **0.54** | **0.39** | - | - | - | **0.66** | **0.52** | 0.17 | **0.63** | **0.37** | **0.26** |
|  | (0.15-2.40) | **(0.21-0.39)** | **(0.37-0.81)** | **(0.19-0.80)** | - | - | - | **(0.47-0.92)** | **(0.37-0.72)** | (0.03-1.05) | **(0.48-0.81)** | **(0.31-0.44)** | **(0.21-0.32)** |
| Black or Black British | **0.07** | **0.27** | 0.53 | **0.24** | - | - | - | **0.45** | **0.46** | 0.39 | 1.04 | **0.30** | **0.33** |
|  | **(0.02-0.34)** | **(0.15-0.48)** | (0.22-1.28) | **(0.07-0.83)** | - | - | - | **(0.27-0.75)** | **(0.24-0.87)** | (0.05-3.28) | (0.67-1.60) | **(0.25-0.36)** | **(0.27-0.42)** |
| Mixed | X | 0.71 | 0.71 | 0.46 | - | - | - | 0.72 | 0.69 | X | 1.10 | **0.48** | **0.35** |
|  | X | (0.36-1.40) | (0.31-1.64) | (0.09-2.38) | - | - | - | (0.41-1.24) | (0.33-1.41) | X | (0.65-1.86) | **(0.36-0.63)** | **(0.25-0.49)** |
| Other Ethnic Groups | 0.21 | **0.34** | 0.63 | 0.33 | - | - | - | 1.04 | 0.67 | **0.03** | 0.83 | **0.33** | **0.26** |
|  | (0.04-1.10) | **(0.18-0.62)** | (0.33-1.19) | (0.11-1.04) | - | - | - | (0.63-1.71) | (0.39-1.16) | **(0.01-0.24)** | (0.50-1.39) | **(0.26-0.43)** | **(0.19-0.35)** |
| **Religion** None vs |  |  |  |  |  |  |  |  |  |  |  |  |  |
| Buddhist | - | - | - | - | - | 1.05 | - | **0.41** | - | X | - | **0.68** | **0.43** |
|  | - | - | - | - | - | (0.29-3.81) | - | **(0.19-0.90)** | - | X | - | **(0.47-0.99)** | **(0.28-0.65)** |
| Christian | - | - | - | - | - | 0.89 | - | **0.65** | - | **0.15** | - | **0.68** | **0.73** |
|  | - | - | - | - | - | (0.56-1.40) | - | **(0.48-0.87)** | - | **(0.03-0.91)** | - | **(0.59-0.78)** | **(0.61-0.87)** |
| Hindu | - | - | - | - | - | 0.60 | - | 0.70 | - | 1.70 | - | **0.73** | 0.97 |
|  | - | - | - | - | - | (0.29-1.27) | - | (0.45-1.10) | - | (0.17-17.36) | - | **(0.59-0.91)** | (0.76-1.24) |
| Jewish | - | - | - | - | - | X | - | 0.82 | - | 1.14 | - | 1.07 | 0.57 |
|  | - | - | - | - | - | X | - | (0.24-2.86) | - | (0.17-7.49) | - | (0.47-2.40) | (0.24-1.34) |
| Muslim | - | - | - | - | - | **0.38** | - | **0.50** | - | 0.09 | - | **0.58** | 0.82 |
|  | - | - | - | - | - | **(0.20-0.72)** | - | **(0.34-0.75)** | - | (0.01-2.13) | - | **(0.48-0.69)** | (0.66-1.02) |
| Other | - | - | - | - | - | 0.48 | - | **0.26** | - | X | - | 0.85 | **0.55** |
|  | - | - | - | - | - | (0.13-1.71) | - | **(0.11-0.63)** | - | X | - | (0.53-1.38) | **(0.33-0.92)** |
| Sikh | - | - | - | - | - | X | - | 0.59 | - | - | - | 0.73 | 1.17 |
|  | - | - | - | - | - | X | - | (0.25-1.41) | - | - | - | (0.53-1.02) | (0.79-1.74) |
| **Sexual Orientation** Heterosexual/ Straight vs |  |  |  |  |  |  |  |  |  |  |  |  |  |
| Bisexual | - | - | - | - | X | - | - | 2.41 | 0.50 | - | - | - | - |
|  | - | - | - | - | X | - | - | (0.30-19.75) | (0.10-2.52) | - | - | - | - |
| Lesbian/ Gay/ Homosexual | - | - | - | - | - | - | - | **0.31** | **0.23** | - | - | - | - |
|  | - | - | - | - | - | - | - | **(0.14-0.70)** | **(0.09-0.61)** | - | - | - | - |
| Other | - | - | - | - | - | - | - | 1.67 | 1.04 | - | - | - | - |
|  | - | - | - | - | - | - | - | (0.18-15.45) | (0.11-9.89) | - | - | - | - |
| **Disability**  No vs |  |  |  |  |  |  |  |  |  |  |  |  |  |
| Yes | - | **0.64** | - | - | - | **0.48** | **0.28** | **0.52** | - | - | **0.65** | **0.43** | **0.59** |
|  | - | **(0.43-0.96)** | - | - | - | **(0.24-0.95)** | **(0.15-0.52)** | **(0.31-0.88)** | - | - | **(0.49-0.86)** | **(0.37-0.51)** | **(0.47-0.73)** |
| **LTFT** No vs |  |  |  |  |  |  |  |  |  |  |  |  |  |
| Yes | - | **-** | - | - | - | **-** | 1.47 | - | - | - | **-** | - | **-** |
|  | - | **-** | - | - | - | **-** | (0.93-2.33) | - | - | - | **-** | - | **-** |
| **Prior Attainment** | - | **1.14** | **1.49** | - | - | **1.30** | **1.23** | **1.38** | **1.23** | **2.00** | **1.35** | **1.56** | **1.19** |
|  | - | **(1.01-1.28)** | **(1.25-1.79)** | - | - | **(1.08-1.57)** | **(1.04-1.47)** | **(1.23-1.54)** | **(1.07-1.42)** | **(1.15-3.46)** | **(1.24-1.48)** | **(1.49-1.65)** | **(1.12-1.27)** |

| **C** | MRCOG Clinical | MRCOG Written | MRCP Clinical | MRCP 1 Written | MRCP 2 Written | MRCPCH Written | MRCPCH Clinical | MRCS Written | MRCS Clinical | RCOphth Ref Cert | MRCPsych Clinical | MRCPsych A Written | MRCPsych B Written |
| --- | --- | --- | --- | --- | --- | --- | --- | --- | --- | --- | --- | --- | --- |
| **N in analysis** | 585 | 2275 | 7350 | 12820 | 8170 | 3535 | 2200 | 5200 | 3005 | 370 | 1845 | 1635 | 1450 |
| **PMQ**  UK vs |  |  |  |  |  |  |  |  |  |  |  |  |  |
| IMG | **0.15** | **0.50** | **0.39** | **0.37** | **0.43** | **0.48** | **0.31** | **0.70** | **0.46** | - | **0.11** | **0.30** | **0.15** |
|  | **(0.06-0.34)** | **(0.39-0.66)** | **(0.33-0.46)** | **(0.33-0.42)** | **(0.36-0.51)** | **(0.38-0.62)** | **(0.22-0.44)** | **(0.56-0.89)** | **(0.34-0.63)** | - | **(0.08-0.15)** | **(0.22-0.40)** | **(0.11-0.21)** |
| **Gender** Males vs |  |  |  |  |  |  |  |  |  |  |  |  |  |
| Females | **2.43** | **-** | **1.21** | **0.67** | **0.77** | 0.86 | **1.57** | **0.55** | **0.77** | - | **1.45** | - | - |
|  | **(1.03-5.74)** | **-** | **(1.10-1.34)** | **(0.62-0.72)** | **(0.68-0.86)** | (0.73-1.02) | **(1.25-1.98)** | **(0.49-0.62)** | **(0.65-0.92)** | - | **(1.11-1.89)** | - | - |
| **Age** ≤29yrs vs |  |  |  |  |  |  |  |  |  |  |  |  |  |
| >29yrs | - | **-** | **0.69** | **0.79** | **0.70** | - | **0.65** | **0.66** | **0.51** | **0.54** | **0.51** | - | **0.68** |
|  | - | **-** | **(0.61-0.79)** | **(0.71-0.89)** | **(0.60-0.81)** | - | **(0.51-0.82)** | **(0.54-0.80)** | **(0.42-0.63)** | **(0.33-0.88)** | **(0.34-0.75)** | - | **(0.49-0.93)** |
| **Ethnicity** White vs |  |  |  |  |  |  |  |  |  |  |  |  |  |
| Asian or Asian British | - | **0.58** | **0.54** | **0.70** | **0.44** | **0.58** | **0.43** | **0.81** | **0.42** | - | **0.35** | - | - |
|  | - | **(0.42-0.79)** | **(0.48-0.61)** | **(0.63-0.79)** | **(0.37-0.52)** | **(0.45-0.74)** | **(0.33-0.55)** | **(0.69-0.96)** | **(0.33-0.53)** | - | **(0.24-0.50)** | - | - |
| Black or Black British | - | **0.53** | **0.52** | **0.58** | **0.53** | **0.57** | **0.37** | **0.60** | **0.47** | - | **0.26** | - | - |
|  | - | **(0.38-0.75)** | **(0.40-0.68)** | **(0.48-0.70)** | **(0.41-0.70)** | **(0.40-0.81)** | **(0.23-0.60)** | **(0.45-0.80)** | **(0.32-0.68)** | - | **(0.16-0.44)** | - | - |
| Mixed | - | 0.70 | **0.71** | 1.01 | **0.68** | 0.93 | 0.60 | 0.90 | **0.56** | - | 0.72 | - | - |
|  | - | (0.44-1.14) | **(0.55-0.92)** | (0.82-1.25) | **(0.50-0.93)** | (0.62-1.39) | (0.35-1.01) | (0.67-1.21) | **(0.36-0.87)** | - | (0.28-1.82) | - | - |
| Other Ethnic Groups | - | 0.87 | **0.55** | **0.76** | **0.60** | 0.80 | 0.67 | **0.72** | **0.55** | - | **0.39** | - | - |
|  | - | (0.55-1.37) | **(0.43-0.69)** | **(0.63-0.93)** | **(0.45-0.79)** | (0.53-1.22) | (0.39-1.16) | **(0.55-0.95)** | **(0.38-0.79)** | - | **(0.19-0.79)** | - | - |
| **Religion** None vs |  |  |  |  |  |  |  |  |  |  |  |  |  |
| Buddhist | - | 1.32 | - | **0.78** | **0.63** | 0.80 | - | 0.98 | 1.04 | - | - | **0.47** | **0.41** |
|  | - | (0.62-2.79) | - | **(0.62-0.98)** | **(0.46-0.87)** | (0.46-1.38) | - | (0.66-1.44) | (0.57-1.91) | - | - | **(0.23-0.97)** | **(0.17-0.99)** |
| Christian | - | **0.73** | - | **0.71** | **0.62** | **0.82** | - | **0.74** | **0.71** | - | - | **0.60** | **0.63** |
|  | - | **(0.58-0.91)** | - | **(0.65-0.78)** | **(0.53-0.72)** | **(0.69-0.97)** | - | **(0.64-0.85)** | **(0.58-0.88)** | - | - | **(0.45-0.79)** | **(0.45-0.88)** |
| Hindu | - | 0.69 | - | **0.82** | **0.65** | 1.12 | - | **0.58** | 0.81 | - | - | **0.42** | 0.65 |
|  | - | (0.44-1.06) | - | **(0.69-0.98)** | **(0.51-0.83)** | (0.79-1.60) | - | **(0.45-0.75)** | (0.57-1.16) | - | - | **(0.27-0.65)** | (0.40-1.07) |
| Jewish | - | 0.92 | - | 0.98 | **0.48** | 0.83 | - | 0.62 | 0.90 | - | - | 0.58 | 0.60 |
|  | - | (0.34-2.48) | - | (0.64-1.50) | **(0.27-0.86)** | (0.42-1.66) | - | (0.30-1.25) | (0.25-3.23) | - | - | (0.18-1.88) | (0.13-2.87) |
| Muslim | - | **0.69** | - | **0.63** | **0.55** | **0.64** | - | **0.50** | **0.65** | - | - | **0.41** | **0.38** |
|  | - | **(0.48-0.99)** | - | **(0.54-0.73)** | **(0.44-0.68)** | **(0.47-0.88)** | - | **(0.41-0.62)** | **(0.49-0.88)** | - | - | **(0.28-0.60)** | **(0.25-0.59)** |
| Other | - | **0.34** | - | 0.83 | 0.72 | 1.11 | - | **0.41** | 0.91 | - | - | **0.42** | **0.27** |
|  | - | **(0.13-0.87)** | - | (0.58-1.17) | (0.43-1.22) | (0.53-2.33) | - | **(0.24-0.70)** | (0.40-2.04) | - | - | **(0.19-0.95)** | **(0.12-0.63)** |
| Sikh | - | 0.86 | - | 0.75 | **0.62** | 1.31 | - | **0.59** | 1.15 | - | - | 1.05 | 1.00 |
|  | - | (0.39-1.89) | - | (0.53-1.07) | **(0.39-0.98)** | (0.62-2.79) | - | **(0.37-0.93)** | (0.54-2.43) | - | - | (0.40-2.76) | (0.30-3.33) |
| **Sexual Orientation** Heterosexual/ Straight vs |  |  |  |  |  |  |  |  |  |  |  |  |  |
| Bisexual | **0.03** | 0.68 | - | 1.21 | - | - | - | - | **0.39** | - | - | **0.42** | - |
|  | **(0.01-0.45)** | (0.34-1.33) | - | (0.85-1.74) | - | - | - | - | **(0.16-0.91)** | - | - | **(0.21-0.86)** | - |
| Lesbian/ Gay/ Homosexual | X | **0.48** | - | **0.75** | - | - | - | - | 0.57 | - | - | 0.72 | - |
|  | X | **(0.29-0.79)** | - | **(0.61-0.92)** | - | - | - | - | (0.31-1.05) | - | - | (0.44-1.16) | - |
| Other | - | X | - | 0.52 | - | - | - | - | 0.70 | - | - | 0.56 | - |
|  | - | X | - | (0.26-1.04) | - | - | - | - | (0.18-2.77) | - | - | (0.18-1.70) | - |
| **Disability**  No vs |  |  |  |  |  |  |  |  |  |  |  |  |  |
| Yes | X | **0.57** | **0.76** | **0.65** | **0.69** | **0.57** | **0.49** | **0.57** | - | - | **0.51** | **0.45** | 0.68 |
|  | X | **(0.40-0.80)** | **(0.60-0.96)** | **(0.56-0.76)** | **(0.54-0.89)** | **(0.44-0.74)** | **(0.33-0.71)** | **(0.46-0.72)** | - | - | **(0.31-0.82)** | **(0.32-0.63)** | (0.44-1.04) |
| **LTFT** No vs |  |  |  |  |  |  |  |  |  |  |  |  |  |
| Yes | - | - | **-** | **-** | - | **-** | **-** | - | - | - | **-** | **-** | - |
|  | - | - | **-** | **-** | - | **-** | **-** | - | - | - | **-** | **-** | - |
| **Prior Attainment** | - | **1.35** | 1.06 | **1.40** | **1.27** | **1.32** | **1.12** | **1.29** | - | **1.29** | **1.24** | **1.47** | **1.45** |
|  | - | **(1.23-1.48)** | (0.99-1.12) | **(1.34-1.46)** | **(1.19-1.36)** | **(1.23-1.42)** | **(1.00-1.24)** | **(1.20-1.37)** | - | **(1.02-1.64)** | **(1.07-1.43)** | **(1.32-1.65)** | **(1.26-1.67)** |

| **D** | Acute Med | Dermatology | Endo & Diabetes | Gastroenterology | Geriatric Med | Nephrology | Palliative Med | Respiratory Med |
| --- | --- | --- | --- | --- | --- | --- | --- | --- |
| **N in analysis** | 385 | 250 | 310 | 265 | 685 | 225 | 220 | 505 |
| **PMQ**  UK vs |  |  |  |  |  |  |  |  |
| IMG | **0.22** | **0.19** | **0.23** | 0.52 | **0.16** | **0.13** | - | **0.36** |
|  | **(0.10-0.46)** | **(0.08-0.46)** | **(0.09-0.60)** | (0.24-1.10) | **(0.07-0.35)** | **(0.04-0.38)** | - | **(0.20-0.65)** |
| **Gender** Males vs |  |  |  |  |  |  |  |  |
| Females | - | - | 1.98 | - | - | **0.43** | - | - |
|  | - | - | (0.95-4.14) | - | - | **(0.19-0.97)** | - | - |
| **Age** ≤29yrs vs |  |  |  |  |  |  |  |  |
| >29yrs | - | - | - | - | X | - | - | X |
|  | - | - | - | - | X | - | - | X |
| **Ethnicity** White vs |  |  |  |  |  |  |  |  |
| Asian or Asian British | **0.25** | - | 0.90 | 0.41 | **0.22** | 0.75 | - | **0.42** |
|  | **(0.10-0.67)** | - | (0.30-2.65) | (0.17-1.01) | **(0.08-0.56)** | (0.28-1.99) | - | **(0.24-0.74)** |
| Black or Black British | **0.17** | - | **0.15** | **0.19** | **0.17** | 1.38 | - | 5.20 |
|  | **(0.05-0.55)** | - | **(0.04-0.57)** | **(0.04-0.83)** | **(0.05-0.67)** | (0.26-7.39) | - | (0.65-41.61) |
| Mixed | 0.26 | - | 0.31 | 0.26 | 0.31 | 0.80 | - | 0.63 |
|  | (0.06-1.18) | - | (0.03-3.29) | (0.05-1.50) | (0.06-1.60) | (0.12-5.35) | - | (0.16-2.49) |
| Other Ethnic Groups | 1.08 | - | 0.47 | **0.10** | **0.16** | **0.07** | - | **0.32** |
|  | (0.18-6.35) | - | (0.12-1.83) | **(0.03-0.44)** | **(0.05-0.58)** | **(0.01-0.40)** | - | **(0.12-0.82)** |
| **Religion** None vs |  |  |  |  |  |  |  |  |
| Buddhist | - | - | - | - | - | - | - | - |
|  | - | - | - | - | - | - | - | - |
| Christian | - | - | - | - | - | - | - | - |
|  | - | - | - | - | - | - | - | - |
| Hindu | - | - | - | - | - | - | - | - |
|  | - | - | - | - | - | - | - | - |
| Jewish | - | - | - | - | - | - | - | - |
|  | - | - | - | - | - | - | - | - |
| Muslim | - | - | - | - | - | - | - | - |
|  | - | - | - | - | - | - | - | - |
| Other | - | - | - | - | - | - | - | - |
|  | - | - | - | - | - | - | - | - |
| Sikh | - | - | - | - | - | - | - | - |
|  | - | - | - | - | - | - | - | - |
| **Sexual Orientation** Heterosexual/ Straight vs |  |  |  |  |  |  |  |  |
| Bisexual | - | - | - | - | - | - | - | - |
|  | - | - | - | - | - | - | - | - |
| Lesbian/ Gay/ Homosexual | - | - | - | - | - | - | - | - |
|  | - | - | - | - | - | - | - | - |
| Other | - | - | - | - | - | - | - | - |
|  | - | - | - | - | - | - | - | - |
| **Disability**  No vs |  |  |  |  |  |  |  |  |
| Yes | **0.23** | - | - | - | - | - | - | - |
|  | **(0.06-0.90)** | - | - | - | - | - | - | - |
| **LTFT** No vs |  |  |  |  |  |  |  |  |
| Yes | - | - | - | - | - | - | - | - |
|  | - | - | - | - | - | - | - | - |
| **Prior Attainment** | **1.61** | - | **2.36** | - | 1.37 | **1.51** | - | **1.62** |
|  | **(1.17-2.22)** | - | **(1.66-3.36)** | - | (0.99-1.87) | **(1.01-2.25)** | - | **(1.25-2.09)** |
