# Supplementary material for: A multivariate analysis to identify the relationship between sociodemographic differences and examination performance in UK postgraduate medical examinations
Source: J R Soc Med. 2025 Nov 3;118(10):325–35. doi: 10.1177/01410768251380980 (PMC12583007; doi:10.1177/01410768251380980)
Supplement: sj-docx-4-jrs-10.1177_01410768251380980. – Supplemental material for A multivariate analysis to identify the relationship between sociodemographic differences and examination performance in UK postgraduate medical examinations [file sj-docx-4-jrs-10.1177_01410768251380980.docx]

**Supplementary Table 4.** Intersectionality of Ethnicity and Religion variables. In total, 144,410 International and UK Medical Graduates had matched ethnicity and religion data, revealing a strong (Spearman’s Rho) correlation of 0.506 (p<0.001). All values are given as percentages (total counts rounded to the nearest 5).

|  | No Religion | Buddhist | Christian | Hindu | Jewish | Muslim | Other | Sikh | Total  (n) |
| --- | --- | --- | --- | --- | --- | --- | --- | --- | --- |
| White | 53.5%  (41480) | <1%  (205) | 43.0%  (33280) | <1%  (-) | 1.2%  (895) | 1.1%  (840) | 1.0%  (775) | 0%  (0) | 77485 |
| Asian or Asian British | 13.2%  (6195) | 6.7%  (3135) | 10.0%  (4670) | 29.7%  (13940) | <1%  10 | 34.9%  (16380) | 1.3%  (620) | 4.2%  (1970) | 46920 |
| Black or Black British | 4.2%  (340) | <1%  (-) | 74.3%  (5980) | <1%  (-) | <1%  (-) | 20.7%  (1670) | <1%  (50) | 0%  (0) | 8050 |
| Mixed | 47.7%  (2305) | 1.6%  (75) | 27.7%  (1340) | 1.1%  (55) | <1%  (25) | 18.2%  (880) | 2.8%  (135) | <1%  (10) | 4830 |
| Other | 14.5%  (1035) | 3.6%  (255) | 17.2%  (1225) | 1.6%  (115) | <1%  (30) | 60.1%  (4285) | 2.2%  (160) | <1%  (15) | 7125 |
